# RT-SHIV, an infectious CCR5-tropic chimeric virus suitable for evaluating HIV reverse transcriptase inhibitors in macaque models

**DOI:** 10.1186/1742-6405-6-23

**Published:** 2009-11-05

**Authors:** Yonghou Jiang, Baoping Tian, Mohammed Saifuddin, Michael B Agy, Peter Emau, J Scott Cairns, Che-Chung Tsai

**Affiliations:** 1 Washington National Primate Research Center, University of Washington, Box 357330 Health Sciences Building, Seattle, Washington 98195, USA; 2 CONRAD, Eastern Virginia Medical School, Arlington, Virginia 22209, USA; 3 Fifth Opus Consulting, LLC Mercer Island, Washington 98040, USA

## Abstract

**Background:**

Non-nucleoside reverse transcriptase inhibitors (NNRTIs) are an important category of drugs for both chemotherapy and prevention of human immunodeficiency virus type 1 (HIV-1) infection. However, current non-human primate (NHP) models utilizing simian immunodeficiency virus (SIV) or commonly used chimeric SHIV (SIV expressing HIV-1 envelope) are inadequate due to the insensitivity to NNRTIs. To develop a NHP model for evaluation of NNRTI compounds, we characterized a RT-SHIV virus that was assembled by replacing the SIV_mac239 _reverse transcriptase (RT) with that of HIV-1HXB2. Since RT-SHIV exhibited *in vitro *characteristics of high infectivity, CCR5-usage, and sensitivity to HIV-1 specific NNRTIs, this virus was thought to be suitable for mucosal transmission and then was used to carry out a vaginal transmission study in pigtail macaques (*Macaca nemestrina*).

**Results:**

RT-SHIV exhibited *in vitro *characteristics of an infectious CCR5-tropic chimeric virus. This virus was not only highly sensitive to HIV-1 RT specific NNRTIs; its replication was also inhibited by a variety of NRTIs and protease inhibitors. For *in vivo *vaginal transmission studies, macaques were either pretreated with a single dose of DMPA (depot medroxyprogesterone acetate) or left untreated before intravaginal inoculation with 500 or 1,000 TCID_50 _of RT-SHIV. All macaques became systemically infected by 2 or 3 weeks post-inoculation exhibiting persistent high viremia, marked CD4^+^T cell depletion, and antiviral antibody response. DMPA-pretreated macaques showed a higher mean plasma viral load after the acute infection stage, highly variable antiviral antibody response, and a higher incidence of AIDS-like disease as compared with macaques without DMPA pretreatment.

**Conclusion:**

This chimeric RT-SHIV has exhibited productive replication in both macaque and human PBMCs, predominantly CCR5-coreceptor usage for viral entry, and sensitivity to NNRTIs as well as other anti-HIV compounds. This study demonstrates rapid systemic infection in macaques following intravaginal exposure to RT-SHIV. This RT-SHIV/macaque model could be useful for evaluation of NNRTI-based therapies, microbicides, or other preventive strategies.

## Background

Heterosexual contact is the predominant route of virus transmission for the HIV epidemics especially in the developing countries worldwide, where women are most vulnerable [1]. The pandemic spread of HIV/AIDS through sexual contact and the slow progress towards an effective vaccine have prompted the search for effective vaginal and rectal microbicides to help mitigate HIV mucosal transmission [2-10]. Various agents have been investigated as topical anti-HIV microbicides including nonnucleoside reverse transcriptase inhibitors (NNRTIs) [2,3,5,11-23]. For an effective preclinical evaluation of these agents, validated animal models are urgently needed. Ideally, the challenge viruses for these models should mimic HIV mucosal transmission predominantly using CCR5 coreceptor, express HIV-1 genes such as RT that are appropriate as therapeutic targets, and induce rapid and readily detectable systemic infection that progress to AIDS-like illness.

NNRTI compounds with high binding affinity for RT are potent inhibitors of HIV-1 replication. However, due to the specific reactive-site requirements of NNRTI, these compounds only inhibit the RT of HIV-1, but not SIV or HIV-2. Thus, while SIV and HIV-2 are well suited to study lentivirus infection and pathogenesis in Asian macaques, they cannot be used to evaluate virus control by HIV-1 specific NNRTI compounds. Early attempts to overcome subtle differences between HIV and SIV while allowing for productive macaque infections resulted in development of several chimeric SHIV strains. The first SHIV construction sought incorporation of HIV-1 env into SIV and was used to challenge macaques immunized with HIV-1 env-based candidate vaccines. After that a number of RT-SHIV strains were constructed to evaluate the activity of HIV-specific NNRTIs both *in vitro *and in macaques [24-29]. Consequently, several macaque models were developed by using different RT-SHIVs [23-26,29-36]. Since most of these RT-SHIV/macaque models were designed to evaluate NNRTIs as therapies, the preferred infection route was intravenous injection. However, recently, mucosal transmission of RT-SHIV have been reported by two laboratories [34,35] in which all rhesus macaques had been pretreated with DMPA (Depo Provera^®^) before intravaginal viral exposure. It is known that prior administration of DMPA enhances mucosal viral transmission by thinning of the vaginal epithelium [37] and also possibly by suppression of antiviral immune response [38].

Clearly, a more physiologically relevant RT-SHIV/macaque model for mucosal transmission will help expedite evaluation of anti-HIV topical microbicides. We have serially passaged an RT-SHIV virus stock obtained from Louis Alexander [28] in different cell types including human and macaque PBMCs before generating a large virus stock in CEMx174 cells for *in vitro *and *in vivo *characterization. The *in vitro *studies show that the new virus stock was highly replicative in both human and macaque cells, predominantly CCR5-tropic and highly sensitive to NNRTIs. This RT-SHIV stock was then used to infect pigtail macaques by intravaginal inoculation. In this report we have demonstrated an efficient transmission of this RT-SHIV through single intravaginal exposure in both DMPA-treated or untreated macaques, and compared the virus transmission efficiency, levels of plasma viremia, clinical outcome and antiviral antibody response between the two groups. Consequently, we have established a suitable non-DMPA RT-SHIV model in pigtail macaques for efficacy evaluation of anti-HIV drugs especially NNRTI-based topical microbicides.

## Results

### *In vitro *replicability of RT-SHIV

**Figure 1:**
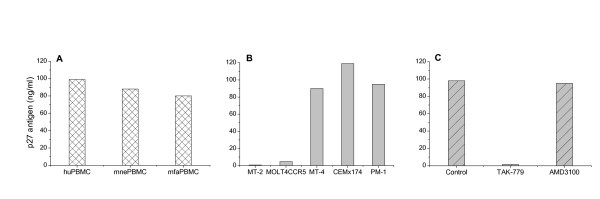
***In vitro *characterization of RT-SHIV**. Replication of RT-SHIV in cultures of PBMCs from humans (huPBMC) and macaques (*Macaca nemestrina*, mnePBMC; *Macaca fascicularis*, mfaPBMC) is shown in panel A. The coreceptor tropism of RT-SHIV is shown in five different human CD4^+^T cell lines in panel B. The specific coreceptor usage by RT-SHIV is shown in panel C where human PBMC cultures were treated with or without coreceptor antagonist TAK-779 or AMD-3100 before incubating with RT-SHIV. Relative virus replication levels (core antigen SIV p27) measured in the culture supernatants after 7 days post incubation, are shown at the left of the panels. Values shown here are representative of three independent experiments.

**Figure 2:**
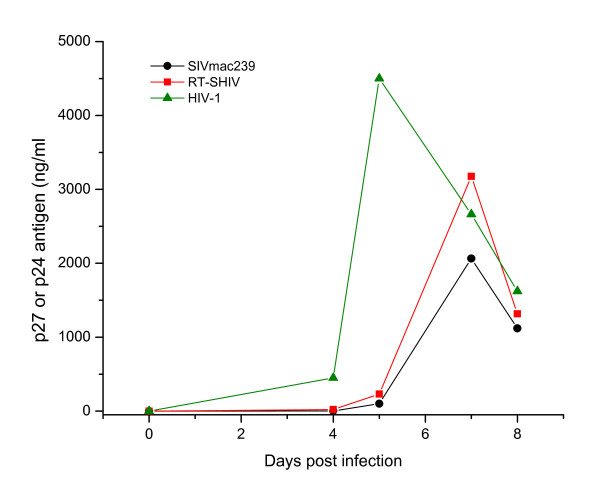
**Replication kinetics of RT-SHIV, SIV, and HIV-1**. Human CEMx174 cells were infected with RT-SHIV, SIV_mac239 _and HIV-1_Lai _at doses equivalent to multiplicity of infection of 0.01. SIV p27 or HIV-1 p24 antigen levels (shown on the left) were measured in culture supernatants to monitor viral replication. Each time point represents the average of duplicate cultures and data shown here are the mean of three independent experiments.

As shown in Figure 1A, RT-SHIV replicated rapidly to high levels in PBMC cultures from both humans (huPBMC) and macaques (*Macaca nemestrina*, mnePBMC and *M. fascicularis*, mfaPBMC). RT-SHIV replication ability in human CD4^+^Tcell lines is shown in Figure 1B. This virus replicated efficiently to high levels in MT-4, CEMx174 and PM1, to low levels in MOLT4CCR5 and poorly in MT-2 cells. The in vitro specificity of CCR5 coreceptor usage by RT-SHIV is shown in Figure 1C. *In vitro *replication kinetics of RT-SHIV, SIV_mac239 _and HIV-1_Lai _in CEMx174 cells are shown in Figure 2. Three viruses exhibited similar replication kinetics as evidenced by the production of viral core antigen in the culture supernatants. Together, these results indicate that RT-SHIV revealed the characteristics of a highly infectious and rapid replicating CCR5-tropic virus.

### Coreceptor usage of RT-SHIV for the entry of target cells

The chemokine receptors CCR5 and CXCR4 are the two main coreceptors essential for HIV, SHIV and SIV entry. To determine chemokine receptor use by RT-SHIV, we studied the ability of five different human CD4^+^T cell lines known to express one or more coreceptors on the cell surface [MT-2 (CXCR4), MT-4 (CXCR4/other), MOLT4CCR5 (CCR5), PM1 (CCR5), and CEM x174 (CXCR4/CCR5/other)] to support replication of this virus. As shown in Figure 1B, the RT-SHIV replicated to a high level in the CCR5-expressing CEMx174 and PM1 cells, and unexpectedly also in MT4 cells that do not express CCR5 but CXCR4 and possibly other receptors. Interestingly, RT-SHIV did not replicate well in MOLT4 cells that express CCR5, and did not grow at all in CXCR4-expressing MT-2 cells. Furthermore, the level of virus replication was directly correlated with the level of syncytium induction (SI) in CCR5-expressing CEMx174 and PM1 cells (syncytial data not shown); however, there was no SI in CXCR4-expressing MT4 cells in spite of high virus replication, and in contrast, relatively high level of SI activity observed in MOLT4 cells with a low level of virus replication. There was no p27 antigen production or SI activity in CXCR4-expressing MT-2 cells (Figure 1B, data not shown). These data may suggest that RT-SHIV predominantly use CCR5 coreceptor for target cell entry; nevertheless other unknown receptors may also be involved for viral entry and SI activity.

Additional evidence of CCR5 coreceptor usage by RT-SHIV was shown in Figure 1C where human PBMC cultures were treated with or without coreceptor antagonists TAK-779 or AMD-3100 before exposing to virus. RT-SHIV replication was markedly inhibited (≥ 91%) by TAK-779 (CCR5 antagonist, 10 μM) [39] but not by a concentration of AMD-3100 (CXCR4 antagonist, 1,000 ng/ml) [40] that strongly inhibited CXCR4-tropic HIV-1_Lai _[41]. Similar results were also obtained with macaque PBMCs (data not shown). Together, these data indicate that our RT-SHIV favors the CCR5 coreceptor mediated entry for replication in the target cells.

### Sensitivity of RT-SHIV and other lentiviruses to NNRTIs

Various concentrations of three different NNRTIs (efavirenz, nevirapine and UC781) were used to assess their inhibitory effects against the replication of HIV-1, RT-SHIV, SIV_mac239_, SIV_mac251_, and SHIV_89.6P _in PBMC and different human CD4^+^cell lines. As shown in Table 1, the 50 percent effective concentration (EC_50_) of each compound against specific virus and their 50 percent cytotoxicity concentration (CC_50_) (tested in uninfected cell cultures) calculated by linear regression analysis were determined in specific target cells. The replication of HIV-1 and RT-SHIV was markedly inhibited by all three NNRTIs, while SIV_mac239_, SIV_mac251_, and SHIV_89.6P _were either not inhibited at all or inhibited to a much lesser extent. These results confirmed that RT-SHIV retained the HIV-1 RT encoding region and remained sensitive to NNRTIs.

**Table 1:** *In vitro *efficacy of NNRTIs against RT-SHIV and other lentiviruses

NNRTIs	CC_50_(μM)	EC_50_(μM)				
		HIV-1*	**RT-SHIV**	**SIV_mac239_**	**SIV_mac251_**	**SHIV_89.6P_**

Efavirenz	3~45	< 0.001	0.003	> 5	> 5	> 5
Nevirapine	84~100	0.028	0.06	> 100	> 100	> 100
UC781	51~66	< 0.005	0.003	> 50	> 10	> 20

CC_50_: 50% cytotoxic concentration; EC_50_: 50% effective concentration.

*NNRTIs showed a strong inhibitory activity to both HIV-1_Lai _and HIV-1_BaL_.

### Inhibitory effects of other anti-HIV compounds on RT-SHIV replication

In addition to NNRTIs, we also measured the inhibitory effects of a variety of other anti-HIV compounds on RT-SHIV replication *in vitro*. CEMx174 cells were incubated with RT-SHIV in the presence or absence of various concentrations of each anti-HIV compound. The CC_50 _and EC_50 _were determined as described above. The selectivity index (SI), the ratio of CC_50 _to EC_50 _was also determined. As shown in Table 1 and Table 2, RT-SHIV was markedly inhibited by all three NNRTIs (UC781, efavirenz, and nevirapine), and also by a number of NRTIs (AZT and D4T), and protease inhibitors (PIs: indinavir and saquinavir). The antiviral effect was less pronounced with 3TC, FTC, tenofovir, and ritonavir; nevertheless, these *in vitro *efficacy data indicate that RT-SHIV is sensitive not only to NNRTIs but also to NRTIs and PIs. Thus, the RT-SHIV/macaque model could be suitable for efficacy evaluation of microbicides and a variety of anti-HIV drugs (i.e., NNRTIs, NRTIs, or PIs).

**Table 2:** Inhibition of RT-SHIV replication by antiretroviral compounds

Drugs Category	Anti-HIV drugs	EC_50_ (μM)	CC_50_ (μM)	Selectivity index(SI)
NNRTIs	Efavirenz	0.002 ± 0.001	35.72 ± 25.45	17860
	Nevirapine	0.06 ± 0.01	> 100	> 1667
	UC781	0.003 ± 0.002	59.22 ± 6.56	19740
NRTIs	ZIdovudine (AZT)	0.01 ± 0.01	> 100	> 10000
	Lamivudine (3TC)	0.43 ± 0.13	> 100	> 233
	Stavudine (D4T)	0.01 ± 0.01	66.48 ± 34.84	6648
	Emtricitabine (FTC)	0.1 ± 0.1	> 100	> 1000
	Tenofovir	0.82 ± 0.62	> 100	> 122
PIs	Ritonavir	0.1 ± 0.1	63.92 ± 43.03	639
	Indinavir	0.008 ± 0.008	> 100	> 12500
	Saquinavir	0.004 ± 0.007	62.18 ± 32.75	15545

NNRTIs: Non-nucleoside reverse transcriptase inhibitors; NRTIs: nucleoside RT inhibitors;

PIs: protease inhibitors

EC_50_: Effective concentration of antiretroviral drug inhibited 50% virus replication.

CC_50_: Cytotoxic concentration of antiretroviral drug showed cytotoxic effect in 50% of cells.

SI: selectivity index represents a ratio of CC_50 _to EC_50_.

### Systemic infection after vaginal RT-SHIV transmission in macaques

**Figure 3:**
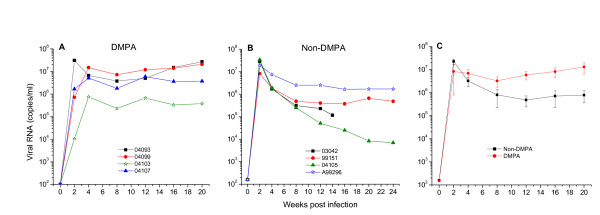
**Plasma viral RNA levels in RT-SHIV infected macaques**. Panel A shows viral RNA (copies/ml) from four macaques that were pretreated with a single 30 mg dose of DMPA (depot medroxyprogesterone acetate, Depo Provera^®^) for 30 days before intravaginal challenge with 500 or 1000 TCID_50 _of RT-SHIV. Panel B shows viral RNA from four macaques that were not pretreated with DMPA before virus challenge. Panel C represents the mean viral loads of four RT-SHIV infected macaques in the DMPA-treated or in the untreated group. In the non-DMPA group, viral RNA data from macaque 03042 were not available after 14 weeks post infection because this macaque was euthanized. The viral load of each plasma sample collected at various weeks pi (x-axis) was tested by standard branched chain DNA amplification assay.

To establish mucosal transmission of RT-SHIV, a group of four pigtail macaques were pretreated with or without DMPA (depot medroxyprogesterone acetate, Depo Provera^®^) for 30 days before inoculating with a single dose of 500 or 1,000 TCID_50 _of virus. Regardless of DMPA pretreatment or the viral inoculum dose (500 or 1,000 TCID_50_), all macaques became systemically infected within two weeks of intravaginal inoculation of RT-SHIV as determined by the presence of both high level viral RNA in plasma (Figures 3A,B,C) and proviral DNA in PBMCs (data not shown). Both DMPA-treated and non-treated macaques showed a peak plasma viremia greater than 10^6 ^copies/ml (5 of 8 macaques had a peak viremia above 10^7^copies/ml) during the acute stage of infection (week 2-4 post inoculation, pi) (Figure 3). However, the set point of virus load at post acute stage (week 8-12 pi) and particularly at the late stage (week 16-20 pi) was significantly higher in DMPA-treated macaques than in non-DMPA macaques (p < 0.05, independent *t *tests).

### Changes of CD4^+^T cell and B cell numbers in RT-SHIV infected macaques

**Figure 4:**
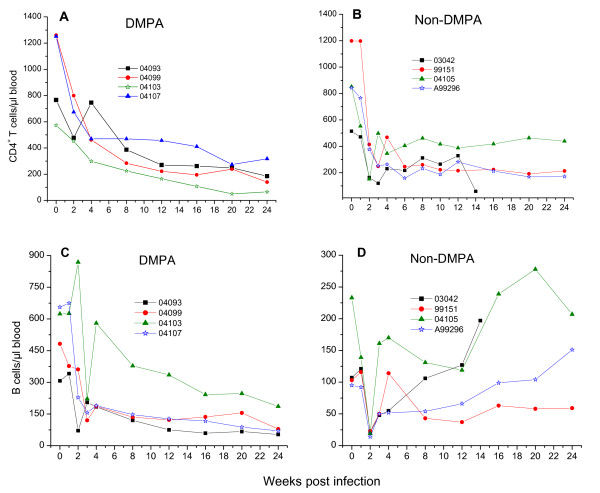
**Changes of CD4^+^T cell and B cell numbers in RT-SHIV infected macaques**. A group of four macaques were either pretreated with a single dose of DMPA or untreated for 30 days prior to intravaginal virus challenge. Whole blood samples collected from macaques were analyzed at various weeks post infection (x-axis) for absolute CD4^+^T cells (panel A, DMPA; panel B, non-DMPA) and B cells (panel C, DMPA; panel D, non-DMPA) numbers that are shown at the y-axis.

All RT-SHIV infected macaques showed a significant CD4^+^T cell depletion in peripheral blood (Figure 4A,B). The decline of CD4^+^T cells at the acute stage of infection (week 2-4 pi) was slightly more pronounce in non-DMPA macaques than the DMPA-treated macaques. As shown in Figure 4C and 4D, both DMPA-treated and untreated macaques also showed a sharp decrease of B cells in peripheral blood during the initial phase of viral infection (week 2-3 pi). Interestingly, three of four DMPA-treated macaques showed a gradual decline of B cells until the end of the study (week 24 pi) but one macaque (04103) showed a large increase in B cells at week 4 pi and then a gradual decline until week 24 pi. In contrast, three of four non-DMPA macaques showed a continuous increase of B cells until week 24 pi, and one macaque (99151) in this group maintained a moderately low level B cell during week 12-24 pi following a slight increase at week 4 pi. The overall lower B cell numbers in DMPA macaques compared to non-DMPA group imply that DMPA treatment may have affected the humoral antiviral immune response.

### Antiviral antibody response in RT-SHIV infected macaques

**Figure 5:**
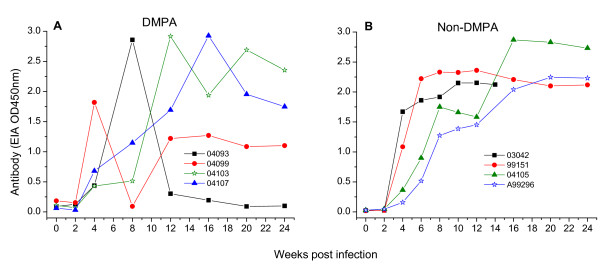
**Antiviral antibody response in RT-SHIV infected macaques**. A group of four macaques were either pretreated with a single dose of DMPA (Panel A) or untreated (Panel B) for 30 days prior to intravaginal virus challenge. Serum samples collected from each macaque at different weeks post infection (x-axis) were tested for anti-SIV antibodies by an enzyme immunoassay. The OD450 nm value of each serum shown at the y-axis is the mean of two different values.

Antiviral antibodies (Abs) were detected in all RT-SHIV infected macaques at week 4 pi (Figure 5). DMPA-treated macaques showed a significant variation in their Ab response. Three macaques (04103, 04093 and 04099) showed greatly fluctuating levels of Ab while macaque 04107 showed gradual increase in Ab up to week 16 pi before decreasing at later time points. In contrast, non-DMPA macaques showed an uniform high level Ab response which increased gradually until peaked between week 12-16 pi, and was maintained thereafter except the macaque A99296 that peaked at week 20 before plateauing.

### Clinical features of RT-SHIV infected macaques

The clinical outcomes were more severe in the DMPA-treated macaques than non-DMPA macaques. Three of four macaques in DMPA group were euthanized due to AIDS-like disease (severe CD4^+^T cell depletion, weight loss, recurrent diarrhea) by week 24 pi. Positive proviral DNA and recoverable infectious virus was detected in blood, lymphoid and reproductive tissues collected at necropsy in these three macaques. The fourth macaque (04107) with persistent viremia and severe CD4^+^T cell depletion also showed AIDS-like disease and was euthanized at 42 weeks pi. Blood, spleen and lymphoid tissues collected at necropsy revealed positive proviral DNA. In the non-DMPA-treated group, one macaque (03042) was euthanized at week 14 pi because of clinical complications with fatty liver, decreased liver function, weight loss, gastroenterocolitis, hind leg immobility, and frequent vocalizing. Severe loss of CD4^+^T cells and moderate level of proviral DNA were detected in peripheral blood, spleen, and lymphoid tissues of this macaque at euthanasia. The severity of disease syndrome of this macaque (03042) may have been only partially associated with RT-SHIV infection. The other three macaques in this group did not develop AIDS-like illness before euthanized after week 24 pi.

## Discussion

The purpose of this study was to thoroughly characterize an RT-SHIV virus and to determine whether this virus can be used to evaluate *in vivo *efficacy of potential HIV-1 preventive or therapeutic strategies especially NNRTI-based topical microbicides. We have shown that RT-SHIV replicates efficiently in both human and macaque PBMCs and also in many different human CD4^+^T cell lines. This virus preferentially utilizes the CCR5 coreceptor for entry into the target cells. The *in vitro *efficacy and cytotoxicity evaluation of NNRTIs and other anti-HIV compounds against RT-SHIV infection were performed by using our virus stock which was originally derived from Louis Alexander [28]. As shown in Tables 1 and 2, this RT-SHIV was sensitive not only to three NNRTIs but also to NRTIs and PIs tested. The *in vitro *antiviral activity of NNRTIs in this study was comparable to other studies [19,24] against the RT-SHIV virus which was originally derived from the same source [23]. In addition, another RT-SHIV_mne _also showed a similar sensitivity to NNRTIs [26] as compared to this study. In short, our RT-SHIV stock is highly sensitive to not only NNRTIs but also to other anti-HIV compounds (e.g., NRTIs and PIs) and thus, is suitable for *in vitro *efficacy evaluation of a wide variety of anti-HIV compounds.

To determine *in vivo *susceptibility of pigtail macaques we conducted a vaginal transmission study with RT-SHIV. All macaques became systemically infected with persistent high plasma viremia within two weeks after a single intravaginal inoculation of either 500 or 1,000 TCID_50 _of virus regardless of DMPA pretreatment. All infected macaques show consistent CD4^+^T cell depletion and positive anti-viral antibody response. There were no discernable differences in virus load and CD4^+^T cell depletion between DMPA- and non DMPA-treated macaques especially during the acute phase of infection (weeks 1-4 pi). However, the average viremic level during the later stage (up to 20 weeks pi) was significantly higher in the DMPA-treated macaques (Figure 3A). Furthermore, the changes in B cell numbers, and the antiviral antibody responses were also significantly different between the two groups (Figures 4 and 5). Following the onset of modest increase during the acute infection (3-4 weeks pi) the B cell numbers in DMPA group continue to decrease until 24 weeks pi. While in contrast, the B cell numbers appear to increase in the non-DMPA group. Similarly, the antibody response was highly variable and overall lower in most of the DMPA macaques compare to the non-DMPA group. Immunosuppression has been noted previously in DMPA-treated macaques [38]. These investigators have also indicated low virus-specific IFN-gamma production in DMPA-treated macaques in response to SIV Gag. The immunosuppressive effect of DMPA on antiviral immune response may at least in part account for the increased viral burden and the diversity in antibody level seen in this study and by others [38]. Nevertheless, our findings with non-DMPA treated macaques indicate that this RT-SHIV virus is highly infectious and readily transmissible through vaginal mucosa in pigtail macaques.

Most of the DMPA-treated animals developed AIDS-like disease compared to non-DMPA macaques. A couple of other laboratories have also reported intravaginal transmission of RT-SHIV in DMPA-treated rhesus macaques [34,35]. In these studies the peak plasma viremia levels were similar to this current study, however, none of the infected macaques developed AIDS-like illness. These studies did not test RT-SHIV for mucosal susceptibility in non-DMPA treated macaques. In any case, regardless of the higher clinicopathologic severity in DMPA-treated macaques, the non-DMPA RT-SHIV model could be considered more relevant for evaluating microbicide or therapeutic agents especially since there will be no systemic effects on immune system as well as no unnatural thinning of vaginal mucosa caused by DMPA. Besides, single exposure of a moderate 500 or 1,000 TCID_50 _dose of our RT-SHIV resulted in infection of all the exposed animals without DMPA treatment, and the peak viremia was similar to DMPA-treated animals observed in this and also in the other two studies [34,35]. Furthermore, careful mucosal infectivity dose titration may determine that even lower amount of virus could infect 100% pigtail macaques as shown in another study where a much lower dose of this virus infected all the intravaginally exposed cynomolgus macaques without DMPA treatment [36].

Nonhuman primates provide a surrogate animal model for HIV/AIDS study since they can not be directly infected by HIV. Moreover, they are also significantly less susceptible to chimeric SHIV (SIV with HIV-1 envelope); therefore, infection of high percentage of macaques through mucosal exposure with SHIV remains to be a challenge. To circumvent this low susceptibility issue, two macaque models; single-dose DMPA and repeated low-dose (non-DMPA) infection models, have been investigated [11,12,42]. DMPA causes non-physiological thinning of vaginal epithelia to enhance virus penetration through mucosa. Also, evidence suggests that DMPA may have global effects on immune system as mentioned earlier [38]. On the other hand, repeated low-dose model may achieve a high rate of macaque infection through large number of mucosal exposures; however, it is proved to be highly labor intensive, expensive and technically challenging. Overall, given the drawbacks of the two existing models, intravaginal susceptibility of 100% macaques by single dose of our RT-SHIV without DMPA-treatment (observed in this study) may prove to be a model of clear advantages over the both single-dose DMPA and repeated low-dose (non-DMPA) macaque models.

## Conclusion

*In vitro *studies confirmed that this RT-SHIV stock exhibits the critical characteristics of high replication capability in PBMCs from both humans and macaques, and the preferential use of CCR5-coreceptor for the viral entry into target cells. *In vitro *antiviral assays also revealed that RT-SHIV is sensitive to HIV-specific NNRTIs and other major classes of anti-HIV drugs. More importantly, *in vivo *study demonstrated that this virus is highly infectious and readily transmissible through vaginal mucosa of macaques, without pretreatment with DMPA (Depo Provera^®^). Thus, this RT-SHIV macaque model may have advantages over the existing models and could be suitable for preclinical efficacy evaluation of NNRTI-based microbicides or other prevention strategies against sexual transmission of HIV-1.

## Materials and methods

### Viruses

The parental chimeric RT-SHIV virus (RT/SHIV/TC) kindly provided by Louis Alexander [28] was initially propagated in CEMx174 cells and tested in several *in vitro *studies using human lymphoid cell lines and PBMCs. To increase infectivity, this virus was serially passaged in CEMx174 cells and PBMCs from both healthy humans and Asian-origin macaques. Since this RT-SHIV replicates better in CEMx174 cells than in phytohemagglutinin (PHA)-stimulated PBMCs (Figure 1A,B), a large stock of actively propagating viruses (named RT-SHIV) was prepared in CEMx174 cells. The infectivity titer of this virus stock was also determined in CEMx174 cells as10^4^TCID_50_/ml. SHIV_89.6P _was originally obtained from Keith Reimann [43] and propagated in PHA-stimulated PBMC from naïve pigtail macaques. HIV-1_Lai_, HIV-1_BaL_, SIV_mac239 _and SIV_mac251_were obtained from the NIH AIDS Research and Reference Reagent Program. SIV strains were also propagated and titrated in CEMx174 cells.

### Cells and reagents

Human cell lines (CEMx174, MT-2, MT-4, PM-1, MOLT4CCR5) and all anti-HIV compounds (Efavirenz, Nevirapine, UC781, AZT, 3TC, D4T, FTC, Tenofovir, Ritonavir, Saquinavir, TAK-779, and AMD-3100) were obtained through the NIH AIDS Research and Reference Reagent Program. Cell lines were maintained in complete HEPES-buffered RPMI-1640 medium containing 10% heat-inactivated FBS (fetal bovine serum) (GEMINI Bio-products, Woodland, CA), 2 mM L-glutamine and 50 μg/ml penicillin/streptomycin (GIBCO, Grand Island, NY). PBMCs were obtained from healthy donors of humans or macaques (*M. nemestrina *and *M. fascicularis*). All PBMCs were isolated from EDTA-treated blood of donors by Ficoll gradient centrifugation and cultured for 48 h in RPMI medium containing rIL2 (20 ng/ml) and PHA (2.5 μg/ml) at a density of 2×10^6 ^cells/ml. The stimulated PBMCs were washed free of PHA and cultured in complete RPMI1640 medium containing rIL2 (20 ng/ml), 10% FBS, and 50 μg/ml penicillin/streptomycin.

### *In vitro *characterization of RT-SHIV

To assess virus infectivity, PHA-stimulated PBMCs (humans or macaques) or CEMx174 cells were incubated for 4 hours with RT-SHIV at a multiplicity of infection (moi) of 0.01, washed once, and then cultured for 7-10 days. The infectivity of all other viruses (SIV, SHIV, or HIV-1) was tested as controls following the same procedure. To determine coreceptor usage, triplicate wells containing PHA-activated human or macaque PBMC (10^6^ml) were pretreated with or without the CCR5 antagonist TAK-779 (10 μM) or the CXCR4 antagonist AMD-3100 (1,000 ng/ml) for 2 hours at 37°C incubation before adding to RT-SHIV. Cells were then incubated with RT-SHIV for another four hours, washed to remove unattached virus, and cultured for 7-10 days. Virus replication in cell cultures was monitored by cytopathic effect (syncytium formation)) and by measuring either SIV p27 or HIV-1 p24 antigen in the culture supernatants using ELISA (ZeptoMetrix Corporation, Buffalo, NY).

### *In vitro *efficacy assays for NNRTIs and other anti-HIV compounds

To determine *in vitro *efficacy of NNRTIs or other drugs, target cells (MT-4, CEMx174 and MT-2) were added into 96-well plates (1 × 10^4^cells/well) in duplicate and treated with or without various concentrations of drugs for two hours at 37°C before incubating with different viruses (HIV-1, SIV and SHIV_89.6P_) for 6-7 days. Virus replication was monitored by measuring either SIV p27 or HIV-1 p24 antigen in the culture supernatants by ELISA. This procedure was used to determine the 50% effective concentration (EC_50_) for each drug [44].

### Drug cytotoxicity assays

To determine the cytotoxic effects, actively growing target cells were similarly incubated with increasing concentration of each drug for 6-7 days without adding the virus and then monitored the cell viability. Cytotoxicity was evaluated by the standard MTT [3-(4,5-dimethylthiazol-2-yl)-2,5-diphenyltetrazolium bromide] assay as described previously [45]. This assay was used to determine the 50% cytotoxic concentration (CC_50_) for each drug [44]. All experiments were run in duplicate.

### Macaques and clinical procedures

Eight naïve pigtail macaques (*M. nemestrina*) were housed at the Washington National Primate Research Center in compliance with its *Guide for the Care and Use of Laboratory Animals*. All macaques were confirmed to be serologically negative for simian type D retrovirus, SIV, and simian T-cell lymphotropic virus (STLV) prior to infection. One group of four macaques was pretreated with DMPA (depot medroxyprogesterone acetate, Depo Provera^®^; a single dose of 30 mg) for 30 days prior to virus challenge to minimize animal susceptibility variation by synchronizing their reproductive cycles. Another group of four macaques were not pretreated with DMPA prior to virus challenge. Two macaques in each group were inoculated intravaginally with 500 TCID_50 _of RT-SHIV; the remaining two macaques in each group received 1,000 TCID_50_as previously described [13,14]. Briefly, 0.5 ml of virus preparation was atraumatically introduced to the vaginal mucosa using a stainless steel animal feeding tube. All macaques were kept in a knee-chest position for at least 15 minutes to maintain exposure of the virus inoculum to the cervicovaginal mucosa. To monitor systemic virus infection, blood samples were collected from each macaque at weekly during the first month, then every two weeks for two months and then monthly for next three months pi. All procedures for animal handlings were performed under ketamine (10 mg/kg) anesthesia. All macaques were inspected daily for general health condition and any signs or symptoms of illness. Other clinical assessments included periodic blood samples for CBC (complete blood counts), serum biochemistry tests, and clinical laboratory diagnoses of suspected causes, if indicated.

### Lymphocyte subset analysis

To quantify the absolute number of peripheral blood CD4^+^T cells, CD8^+^T cells and B cells in the infected macaques, lymphocyte phenotyping was performed on EDTA blood samples stained with mouse anti-human monoclonal antibodies that reacted with macaque lymphocytes. Lymphocyte subsets were analyzed with a FACSCalibur flow cytometer and Cellquest software (Becton Dickinson). CD4^+ ^and CD8^+ ^T lymphocytes were defined as CD3^+^CD4^+ ^and CD3^+^CD8^+^, respectively and B lymphocytes as CD3^-^CD20^+^.

### Virus isolation from RT-SHIV infected macaques

The frequency of infected cells in blood was estimated by quantitative coculture of PHA-stimulated PBMC with uninfected CEMx174 cells. RT-SHIV from infected cells readily infects CEMx174 cells and induces marked syncytia in the culture. Infection was monitored by microscopic examination for syncytium formation and by measuring SIV gag protein in the culture supernatants using SIV p27 antigen capture ELISA (ZeptoMetrix Corp., Buffalo, NY).

### Detection of plasma virus RNA

The level of plasma viremia was quantified by the standard branched chain DNA (bDNA) amplification assay (Siemens Medical Solutions Diagnostics, Berkeley, CA). The limit of detection of viral RNA was 125 copies per milliliter of plasma.

### Detection of proviral DNA in PBMC

Genomic DNA was extracted from 1 × 10^6 ^PBMC using the Qiagen DNA Blood Kit (QIAamp DNA Blood Mini Kit, Cat # 51106). SIVmac239 *gag*-specific primers were used to amplify virus DNA by PCR as described previously [13,14,46].

### Antiviral antibody assay

Viral antibody titer was assayed by the HIV-2 EIA kit (Bio-Rad Laboratories, Redmond, WA), taking advantage of the strong cross reactivity of SIV antibodies to HIV-2 antigen. Plasma samples from all time points were tested for the presence of RT-SHIV antibodies following manufacturer's protocol.

## Competing interests

The authors declare that they have no competing interests.

## Authors' contributions

YJ carried out the experiments, performed data analysis and wrote the initial draft of the manuscript; BT performed efficacy assays for NNRTIs and other anti-HIV drugs and data interpretation; MBA helped with data interpretation and manuscript review; PE helped with laboratory experiments and data analysis. MS and JSC both supported the study plan for the development of an intravaginal infection model with RT-SHIV, helped with discussion on the study progress and review of manuscript. CCT was responsible for the overall experimental design, implementation of the project, data interpretation and manuscript writing.
